# Genetic Determinants of Tau-PET Imaging in Alzheimer’s Disease: A Narrative Review of Genome-Wide and Candidate-Gene Studies

**DOI:** 10.3390/jimaging12090423

**Published:** 2026-09-08

**Authors:** Jaskeerat Gujral, Om H. Gandhi, Tejas Bhogale, Amir A. Amanullah, Ashil Srivastava, Shashi B. Singh, Thomas J. Werner, Poul Flemming Høilund-Carlsen, Abass Alavi

**Affiliations:** 1 Department of Radiology, Hospital of the University of Pennsylvania, Philadelphia, PA 19104, USA; 2 Yale Department of Radiology and Biomedical Imaging, Yale New Haven Hospital, New Haven, CT 06510, USA; 3 Department of Nuclear Medicine, Odense University Hospital, DK-5000 Odense, Denmark; 4 Department of Clinical Research, University of Southern Denmark, DK-5000 Odense, Denmark

**Keywords:** Alzheimer’s disease, tau, positron emission tomography, genome-wide association study, flortaucipir, endophenotype, narrative review

## Abstract

Alzheimer’s disease (AD) is characterized by extracellular β-amyloid (Aβ) plaques and intracellular hyperphosphorylated tau neurofibrillary tangles, with tau pathology correlating more strongly than amyloid pathology with cognitive decline and neuronal loss. While traditional genome-wide association studies (GWAS) have identified loci associated with tau-related phenotypes in cerebrospinal fluid, these approaches cannot capture the regional brain patterns of tau deposition that may underlie disease heterogeneity. Recent GWAS incorporating tau-PET imaging have begun to address this gap, identifying novel genetic variants associated with tau-PET signal through chromatin modification, microtubule dynamics, and immune regulation. Some of these mechanisms may operate at least partly independently of established amyloid-related pathways. This narrative review synthesizes the peer-reviewed evidence from genome-wide and candidate-gene association studies of tau-PET in AD, classifying findings by biological mechanism to characterize the reported genetic determinants of regional tau-PET signal, to grade the strength of the evidence behind each, and to consider their clinical applications. Throughout, the phenotype under analysis is tracer uptake and not directly measured neuropathological tau, and each locus is interpreted with that constraint in view. Transcriptional regulators such as *ZBTB20* and *EYA4* have been associated with tau-PET signals, although both findings rest on a single, unreplicated analysis. Common *MAPT* variants showed only nominal associations, whereas the *CYP1B1*-*RMDN2* locus, which influences microtubule dynamics and oxidative stress, reached genome-wide significance and is the only locus to date that has been formally replicated in independent cohorts. *CYP1B1* is notable because it offers a plausible mechanism linking oxidative stress to tau aggregation. Genes controlling tau phosphorylation, such as *PPP2R2B* and *FYN*, have also been implicated, although the *PPP2R2B* association was reported without a replication sample and the *FYN* association was nominal (*p* = 0.048) in a candidate-gene analysis of 146 participants. Immune- and trafficking-related loci, including *CR1*, *CLU*, and *BIN1*, suggest contributions of neuroinflammation and endosomal transport to regional tau accumulation. Lastly, *APOE* ε4 is the most frequently reported predictor of tau-PET burden across brain regions, but its effect attenuates in population-based, predominantly cognitively unimpaired cohorts, indicating that it may be substantially amyloid-conditional, not amyloid-independent. Across these studies the genetic architecture of tau-PET signal appears at least partly separable from that of clinically diagnosed AD, although this remains preliminary: no formal genetic correlation has been estimated, the contributing cohorts overlap substantially, and the samples are small enough that a null result carries little weight. The evidence supports continued study of tau-PET as an endophenotype, and it also indicates that the therapeutic implications of these loci are less direct than these studies generally claim.

## 1. Introduction

Alzheimer’s disease (AD) is the leading cause of dementia in individuals over 65 years of age, with global incidence and prevalence continuing to increase over recent decades [1,2,3]. This progressive neurodegenerative disorder impairs memory, executive function, language, visuospatial skills, and behavior [4,5]. Early identification through neuropsychological testing, cerebrospinal fluid biomarkers, and neuroimaging modalities such as magnetic resonance imaging (MRI) and positron emission tomography (PET) may enhance diagnostic accuracy and enable timely intervention [4,5].

AD pathology is accompanied by the accumulation of extracellular β-amyloid (Aβ) plaques and intracellular hyperphosphorylated tau neurofibrillary tangles (NFTs), with tau pathology emerging as a stronger correlate of cognitive decline and neuronal loss than amyloid pathology [6,7,8]. While tau pathology is associated with neurodegeneration and clinical decline, its precise role in AD remains debated. Tau may act as a driver of disease progression, a downstream mediator of other pathological processes, or a marker of neuronal injury. Distinguishing among these possibilities remains an important challenge in AD research.

In AD, tau loses its microtubule-binding function and accumulates as aggregates, and the underlying cellular mechanism contributing to AD pathology involves disruption of cytoskeletal regulation and synaptic dysfunction, although the extent to which tau aggregation directly drives neurodegeneration remains debated [9,10,11]. Six tau isoforms arise from alternative splicing of the microtubule-associated protein tau (*MAPT*) gene, and under normal conditions, these tau proteins stabilize the neuronal cytoskeleton [9,12]. The development of tau-PET imaging using tracers, such as flortaucipir, has enabled in vivo estimation of aggregated tau burden, revealing unique regional deposition patterns that are believed to correlate with cognitive decline and provide insight into disease progression [13]. However, important questions remain regarding tracer specificity, pathological validation, and the interpretation of PET signal as a surrogate for underlying tau pathology.

AD demonstrates substantial heritability (58–79%), motivating genetic studies to identify pathogenic mechanisms underlying tau accumulation [14]. For instance, the apolipoprotein E (*APOE*) ε4 allele influences both amyloid and tau pathology, though its effects on tau may be partially independent of amyloid burden [15,16,17]. While traditional genome-wide association studies (GWAS) have identified loci associated with tau-related phenotypes in cerebrospinal fluid, these approaches cannot capture the regional brain patterns of tau deposition that may underlie disease heterogeneity [18].

Recent GWAS incorporating tau-PET imaging have begun addressing this gap, identifying novel genetic variants associated with tau-PET signal through chromatin modification, microtubule dynamics, and immune regulation, mechanisms that may operate at least partly independently of established amyloid-related pathways [19,20,21,22,23]. Tau-PET is now used to stage disease, to select participants for trials, and as an outcome measure, so what drives variation in the signal bears directly on how imaging studies are designed and interpreted. This narrative review synthesizes the peer-reviewed evidence from genome-wide and candidate-gene association studies of tau-PET in AD, and it addresses four questions: (1) Which genetic variants have been reported to influence regional tau-PET signal, and through which biological processes? (2) How strong is the evidence behind each of them once replication status and cohort overlap are taken into account? (3) How far can an association with tracer uptake be read as an association with tau pathology? (4) How far does the genetic architecture of tau-PET signal overlap with that of clinically diagnosed AD? The included studies and the loci they report are summarized in Table 1; their cohort composition, sample overlap, replication status, and evidence tier are summarized in Table 2; and the methodological features that differ across them, including tracer, phenotype definition, ancestry, covariates, and significance threshold, are summarized in Table 3.

## 2. Methods

This is a narrative review. It was not registered and it does not follow the Preferred Reporting Items for Systematic Reviews and Meta-Analyses (PRISMA), because the aim was to synthesize and grade a small and methodologically heterogeneous body of imaging-genetics studies and not to pool effect estimates. The search strategy, the eligibility criteria, the appraisal approach, and the grading scheme are set out below so that the scope of the review can be assessed. The manuscript was checked against the Scale for the Assessment of Narrative Review Articles (SANRA) during revision [25].

PubMed, Embase, Web of Science, and Google Scholar were searched from database inception to August 2026. The PubMed search string was: (“Alzheimer Disease” [MeSH] OR Alzheimer*) AND (“tau PET” OR “tau positron emission tomography” OR flortaucipir OR AV-1451 OR MK-6240 OR RO948 OR PI-2620 OR THK5351) AND (“Genome-Wide Association Study” [MeSH] OR GWAS OR “candidate gene” OR “gene-based” OR polygenic OR “Mendelian randomization” OR “single nucleotide polymorphism”). Equivalent syntax was used in the other databases. Candidate-gene, gene-based, and Mendelian randomization analyses were sought deliberately through the terms above and were not encountered opportunistically. Reference lists of all included reports and of previous reviews of Alzheimer imaging genetics were hand-searched, and forward citation tracking was carried out in Google Scholar.

An investigation was eligible if it tested an association between germline genetic variation and a tau-PET measure in living human participants, whether by genome-wide, gene-based, candidate-gene, or Mendelian randomization analysis. Reports were excluded if the only tau phenotype was measured in cerebrospinal fluid or plasma, if the only imaging phenotype was amyloid-PET, if no genetic data were analyzed, or if the work concerned tracer development or neuropathological validation without a genetic component. Conference abstracts, editorials, commentaries, and case reports were excluded. Only peer-reviewed reports written in English were included. Preprints were excluded so that every finding graded in Table 1, Table 2, Table 3 and Table 4 has passed peer review; one preprint reporting additional tau-PET loci was identified during screening and is noted in Section 13. No non-English report meeting the remaining criteria was identified, so the language restriction did not exclude an eligible study.

Since the included studies are genetic association studies and not diagnostic-accuracy or intervention studies, risk of bias was appraised narratively against the features that determine how much weight an imaging-genetics association can carry: sample size, ancestry composition, overlap with the cohorts analyzed in the other included studies, the covariates modeled, in particular amyloid burden and *APOE* ε4, the multiple-testing threshold applied, and whether an independent replication sample was analyzed. These features are reported for every included study in Table 2 and Table 3, so that a reader can see the basis for each appraisal and not only its conclusion.

Each reported association was then assigned to one of four evidence tiers. Tier 1 denotes a genome-wide significant association replicated in a sample independent of the discovery cohort. Tier 2 denotes a genome-wide significant association that has not been replicated in an independent sample. Tier 3 represents an association that reached significance within a pre-specified candidate-gene or gene-based analysis without genome-wide testing, after correction for multiple testing where more than one variant was tested. A pre-specified single-variant test is graded here rather than at Tier 4, because the hypothesis was stated in advance, which an uncorrected nominal result in a multi-variant analysis does not require. Tier 4 represents a nominal association only, meaning *p* < 0.05 without correction. Tiers are assigned to individual results and not to studies, so a study reporting results at more than one tier appears at more than one tier, and the same two criteria are applied to genome-wide, gene-based, candidate-gene, and nominal findings alike. Independence is judged at the level of participants and not of cohort names, so a replication sample that contributes participants to another study in this review is not treated as independent.

This scheme was created for this review. It does not map onto GRADE, the Oxford Centre for Evidence-Based Medicine levels of evidence, or any other established grading system [26,27]. Those systems grade the certainty of evidence for a clinical effect across a body of studies, and they turn on constructs such as directness, imprecision relative to a decision threshold, and risk of bias in allocation, none of which has a clear analogue for a genetic association with an imaging phenotype. In this literature, the two properties that determine how much confidence a locus deserves are the significance threshold it crossed and whether it held up outside the sample in which it was found, so the tiers are defined on those two axes and on nothing else. The cost of that simplicity is that the tiers carry no information about effect size, biological plausibility, or the quality of any supporting functional work, each of which is discussed for the individual loci in the text.

## 3. Biological Significance of Tau Pathology: Cause, Correlate, or Consequence

NFTs are a characteristic neuropathological feature of AD and exhibit stronger correlations with cognitive impairment and neurodegeneration than amyloid burden in many studies [28]. However, the presence of such correlations does not establish causality. Three major interpretations of tau pathology have been proposed [29]. First, tau may function as a direct driver of neurodegeneration, whereby tau aggregation contributes to synaptic dysfunction, neuronal injury, and progressive cognitive decline [29]. Experimental models demonstrating tau propagation across interconnected brain regions provide support for this hypothesis [30,31]. Second, tau may act as a downstream mediator of other pathological processes, including amyloid accumulation, neuroinflammation, vascular dysfunction, metabolic impairment, or age-related neuronal stress [29]. Under this framework, tau contributes to disease progression but is not necessarily the initiating pathological event [29]. Third, tau may be a marker of neuronal injury or impaired cellular homeostasis, not a primary disease-causing agent [29]. Consistent with this possibility, substantial tau pathology may be observed in some cognitively unimpaired individuals, while clinical manifestations of dementia do not always correlate directly with the amount of tau detected by biomarkers [28].

Consequently, although tau is strongly supported as a correlate of disease severity and progression, its position within the causal pathway remains unresolved: it may act as a primary driver, a downstream mediator, or a byproduct of neurodegeneration. This matters for the genetic findings reviewed below. A variant that raises regional tau burden is a candidate therapeutic target only under the first interpretation. Under the second and third it marks a process that is already downstream of whatever initiates the disease, which is one reason the loci described here support weaker therapeutic conclusions than the primary studies claim.

## 4. What Tau-PET Measures, and What That Implies for Genetic Association Studies

Every association reviewed below uses tau-PET tracer uptake as the phenotype. Tracer uptake is a composite measurement, which reflects the density of the intended target, the affinity of the tracer for that target, the presence of other tissue constituents that bind the tracer, regional atrophy and the partial-volume effects that follow from it, and the reference region chosen for scaling. Therefore, a genetic variant can shift the tau-PET phenotype by acting on tau aggregation or by acting on any of the other contributors, and an association statistic on its own does not separate these possibilities.

Validation evidence is strongest for Alzheimer-type paired helical filament (PHF) tau, where commonly used tracers show cortical uptake patterns that broadly correspond to advanced Braak-stage neurofibrillary pathology [32,33]. The relationship between PET signal and pathology is less well established in early disease and in non-Alzheimer tauopathies, so tracer uptake should not be read as a direct or universal measure of tau burden [34,35].

Off-target binding is the most direct threat to the phenotype. First-generation tracers, particularly flortaucipir and the THK compounds, show non-specific retention in the basal ganglia, midbrain, choroid plexus, meninges, and skull [36,37,38]. Proposed contributors include neuromelanin-containing regions, iron-associated tissue, calcification, vascular structures, and monoamine oxidase enzymes, so elevated signal in these regions may reflect tracer chemistry or local tissue composition and not neurofibrillary tangles [32,35,36,37,38,39]. Some of these tissue characteristics can be measured directly instead of being left as unmeasured confounding: regional mineralization, for instance, is quantifiable in vivo with [^18^F]NaF PET, including within intracranial structures [40]. Second-generation tracers, including MK-6240, RO948, and PI-2620, were designed to improve selectivity for PHF tau, but off-target uptake, spill-in from adjacent structures, and tracer-specific regional artifacts continue to influence quantification with them as well [34,39]. The size of the difference between tracer generations became clearer with the approval of MK-6240 in August 2026, the second tau-PET agent licensed in the United States [41]. In a prospective within-participant head-to-head comparison, MK-6240 identified neocortical tau pathology more than twice as often as flortaucipir among cognitively unimpaired participants, and in 28% of cognitively impaired participants compared with 16% for flortaucipir [42]. Two tracers that disagree to this extent about who is tau-positive are not interchangeable measures of one quantity. This is the most direct evidence available that a phenotype defined by flortaucipir uptake is not simply tau burden, and every association reviewed here was discovered with flortaucipir, and only one has been tested in a cohort scanned with a chemically distinct tracer. The implication for imaging genetics is that a variant that alters the abundance or distribution of an off-target binding substrate will behave in a GWAS exactly as a variant that alters tau aggregation, and the two can be separated only by evidence obtained outside the PET measurement itself.

Tau-PET also has limited sensitivity for the earliest AD pathological changes. NFTs may arise in small transentorhinal and entorhinal regions before they can be reliably detected, and these regions are susceptible to motion and partial-volume effects and lie close to the choroid plexus, so a negative scan does not exclude early or low-level Alzheimer-type tau pathology [43]. Available imaging-neuropathology evidence indicates that current tracers detect Alzheimer tau more reliably at intermediate and advanced Braak stages, which makes tau-PET more informative for staging than for identifying early molecular change [33,34]. The approved label for MK-6240 puts the same limitation in regulatory language: a negative scan does not necessarily exclude tau NFT pathology, a positive scan does not necessarily confirm it, and performance may be lower in patients at earlier stages of the pathological spectrum [41]. For genetic analysis this produces a restriction of range at the low end of the phenotype, which reduces power to detect variants acting early in the accumulation process.

The phenotype is also biologically distinct from the fluid tau measures against which it is often validated. Cerebrospinal fluid and plasma phosphorylated tau reflect soluble tau phosphorylation and secretion, whereas tau-PET is intended to measure insoluble aggregated PHF tau within neurofibrillary tangles. These are different molecular species produced by different processes, and they change on different schedules, with soluble phosphorylated tau moving earlier in the disease course than aggregated tau detectable by PET [44,45]. Correlations between them should therefore not be read as evidence that they are interchangeable, or that either independently validates the other [46,47,48]. The same caution applies to the validation logic used in this field. If tau-PET is treated as valid because it correlates with elevated CSF phosphorylated tau, while CSF phosphorylated tau is treated as valid because it predicts tau-PET positivity, the agreement between them may reflect a shared association with disease stage and not independent confirmation that either measures the process it is intended to measure. A practical consequence for this review is that concordance between a tau-PET locus and a fluid p-tau measurement is supportive but partial evidence, and that loci identified by GWAS of cerebrospinal fluid tau should not be expected to coincide with loci identified by GWAS of tau-PET.

Hence, first, a genome-wide significant association with tau-PET establishes an association with tracer uptake and not, by itself, with tau pathology. Second, replication in a cohort scanned with a chemically distinct tracer is the most direct available test of a tracer-driven artifact, because a variant acting on binding affinity should not survive the switch; it is a stronger form of replication than a larger sample using the same tracer. Third, orthogonal molecular evidence, such as expression and methylation quantitative trait loci, cell-type-specific expression, fluid biomarker associations, and expression trajectories in tau-model as opposed to amyloid-model animals, does the work that a PET association cannot do by itself. One locus in this review is supported on all three counts, and it is discussed in Section 6.

## 5. Transcriptional and Chromatin Regulation

Genes involved in transcriptional regulation and chromatin state are among the most prominent signals to emerge from tau-PET GWAS. *ZBTB20* is a transcriptional repressor and *EYA4* is a tyrosine phosphatase that also acts as a transcriptional coactivator, so the two should not be described collectively as histone-modifying enzymes, and they do not necessarily converge on one molecular mechanism [49,50,51,52,53]. One GWAS has reported genome-wide significant loci in this category, both from the same ADNI-based sample, and neither has been tested in an independent cohort [19].

Guo et al. performed a GWAS of brain tau load as measured by [^18^F]flortaucipir PET in 543 non-demented European individuals from the ADNI cohort (a sample that overlaps substantially with the ADNI participants analyzed by Stage et al. and Franzmeier et al., and with the ADNI contribution to the discovery meta-analysis of Nho et al.), including participants across the cognitive spectrum from normal cognition to mild cognitive impairment [19]. The study identified two novel genome-wide significant associations with elevated tau deposition: rs56298435 within *ZBTB20* (*p* = 8.35 × 10^−10^, β = 0.31) and rs150532 near *EYA4* (*p* = 1.90 × 10^−8^, β = 0.26), with *APOE* additionally reaching genome-wide significance when *APOE* ε4 was not included as a covariate, which is expected by construction and provides no independent evidence of an *APOE* effect. *ZBTB20* encodes a zinc finger and BTB domain-containing transcriptional repressor involved in neuronal development and hippocampal formation, functioning through chromatin remodeling and transcriptional regulation [54,55]. *EYA4* functions as a dual-activity protein possessing both tyrosine phosphatase activity and transcriptional coactivator function, interacting with SIX-family transcription factors to regulate gene expression programs [56]. Risk allele carriers for rs56298435 and rs150532 exhibited elevated tau SUVRs particularly in temporal and parietal cortices, with phosphorylated tau in cerebrospinal fluid and plasma showing the same direction of effect, which is supportive but is not independent validation [19]. Guo et al. demonstrated that tau-PET statistically mediated the association between rs56298435 and rs150532, respectively, and cognitive decline, while no direct genetic effects on Mini-Mental State Examination score were observed, indicating that tau burden may be the primary pathway linking these variants to cognitive impairment [19].

These loci suggest that altered chromatin states may create permissive environments for tau misfolding and aggregation, though the direction of causality remains to be established. Dysregulated chromatin remodeling could either promote tau pathology through altered gene expression patterns, or conversely, tau accumulation might induce secondary chromatin modifications as a downstream consequence of neurodegeneration. Both loci come from a single ADNI-based analysis, neither has been replicated, and the two genes do not share a molecular mechanism. Neither locus has been examined for the phenotype-level alternatives set out in Section 4: no expression or methylation quantitative trait locus data, no fluid biomarker association independent of the same participants, and no cross-tracer replication have been reported for either variant. These findings are therefore hypothesis-generating and do not establish chromatin remodeling as a mechanism in tau pathology. Further mechanistic studies incorporating temporal analyses and functional validation are warranted to establish whether chromatin modifications are causal drivers of tau pathology or secondary responses to neurodegeneration.

## 6. Microtubule Dynamics and Oxidative Stress

Ramanan et al. performed GWAS using data from a sample of 754 individuals aged greater than 50 years from the Mayo Clinic Study of Aging [22]. Their study investigated the role of microtubule-associated protein tau (*MAPT*). *MAPT* encodes the tau protein, and variation at this locus has been associated with AD. AV-1451 (^18^F-flortaucipir) tau-PET imaging was used to measure the effects of *MAPT* variants on tau accumulation using meta-region of interest (ROI) SUVR. Among the 31 *MAPT* variants analyzed by Ramanan et al., three SNPs demonstrated nominal associations with tau-PET burden, but none met the Bonferroni-corrected significance threshold of *p* < 6.58 × 10^−4^ used in the study’s analysis (0.05/76 variants: 31 *MAPT* variants, 43 established AD risk variants, and the *APOE* ε2 and ε4 alleles). rs1467967, a common variant of the *MAPT* H1 haplotype, was among the three nominally associated variants but did not survive correction for multiple comparisons. Collectively, there is no strong evidence that common *MAPT* variants contribute substantially to tau-PET burden when the Bonferroni-corrected threshold is applied, suggesting that their overall effect is likely minimal.

**Figure 1:**
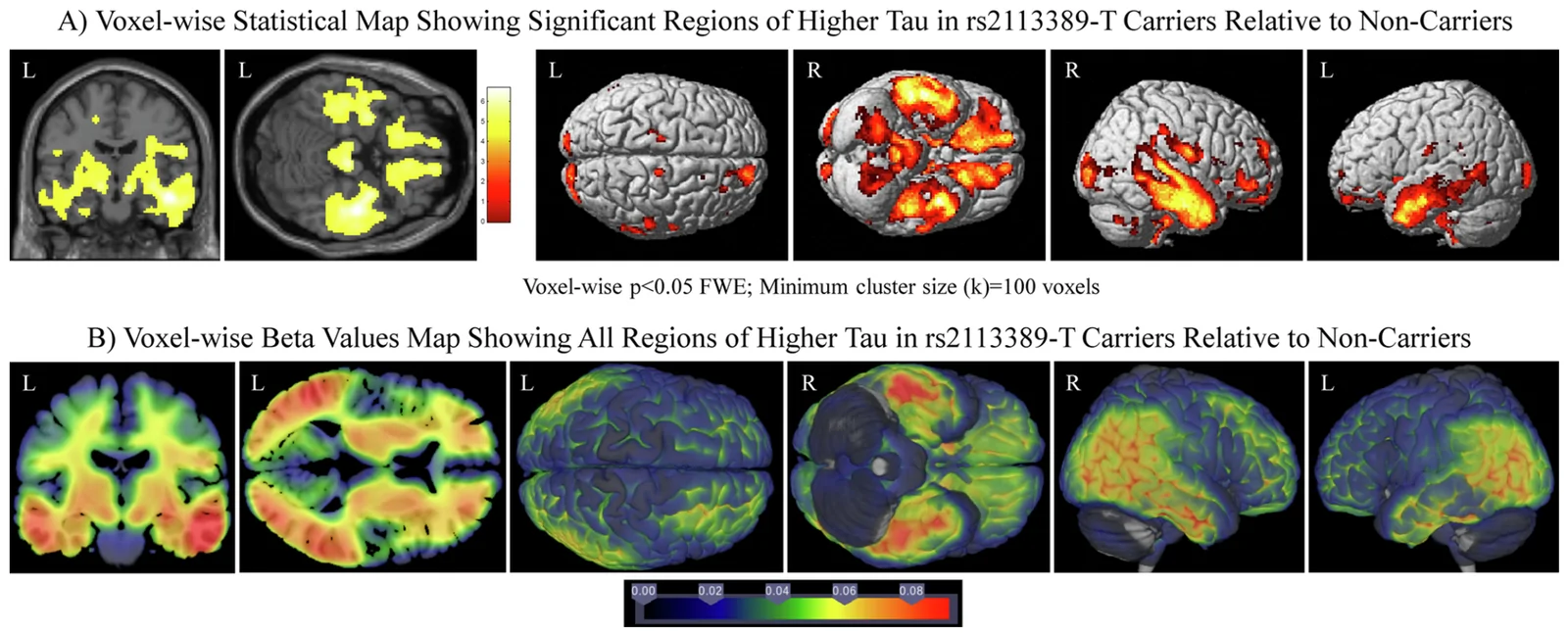
(**A**) Voxel-wise analyses show a widespread association between the rs2113389 dominant genotype and tau deposition in the inferior frontal, parietal, and medial and lateral temporal lobes. Individuals carrying one or more minor T alleles (CT/TT) show greater tau burden than CC homozygotes. Maps are displayed at a voxel-wise threshold of *p* < 0.05 with family-wise error corrections and a minimum cluster size (k) = 100 voxels. (**B**) Beta-value maps show widespread regional trends of greater tau deposition in rs2113389-T carriers relative to non-carriers. Temporal, parietal, and frontal lobe tau is greater in minor allele carriers than their counterparts. Statistical analysis used a one-way analysis of covariance (ANCOVA) model controlling for age, sex, diagnosis, apolipoprotein E (*APOE* ε4) carrier status, and amyloid-beta (Aβ) positivity. Adapted from Nho, K. et al. *CYP1B1*-*RMDN2* Alzheimer’s disease endophenotype locus identified for cerebral tau PET. Nat Commun. September 2024, 15(1):8251 with permission under Creative Commons Attribution 4.0 International Public License (https://creativecommons.org/licenses/by/4.0/legalcode) [21].

Beyond *MAPT*, Nho et al. identified rs2113389 at the *CYP1B1*-*RMDN2* locus, which achieved genome-wide significance (*p* = 1.37 × 10^−8^) across their seven-cohort discovery meta-analysis of 1446 individuals [21]. The association was subsequently replicated in five additional cohorts (*N* = 1600) with a concordant direction of effect, bringing the total sample to 3046 participants across twelve cohorts; this remains the only tau-PET GWAS locus with formal replication in an independent sample. The lead variant showed consistent effect directions across all discovery cohorts with low heterogeneity (I^2^ = 27.8%, *p* = 0.217), and voxel-wise analyses revealed widespread effects in frontal, parietal, and temporal regions. rs2113389 was reported to account for 4.3% of the estimated variance in cortical tau in ADNI, compared with 3.6% for the *APOE* ε4 variant (rs429358). Voxel-wise statistical maps from Nho et al. show greater tau-PET uptake in rs2113389-T carriers across widespread cortical regions, predominantly frontal, parietal, and temporal (Figure 1).

*CYP1B1* encodes cytochrome P450 1B1, an enzyme involved in pathways regulating oxidative stress. It is highly expressed in brain fibroblasts and excitatory neurons and has been described to be upregulated in AD [21]. Oxidative stress induces tau hyperphosphorylation and aggregation through multiple kinase activation pathways, including GSK3β, CDK5, and MAPK cascades [57,58]. Regulator of Microtubule Dynamics 2 (*RMDN2*) showed significant downregulation in the parahippocampal gyrus of AD patients (*p* = 0.004) and encodes a protein that has been shown to regulate microtubule dynamics, though its specific molecular mechanisms and interactions with cytoskeletal components require further functional characterization [21]. The anatomical specificity of these associations, with effects in temporal, parietal, and frontal regions vulnerable to tau pathology, suggests that genetic variants in this locus influence regional patterns of tau deposition across AD-relevant brain networks. This locus is further supported by orthogonal functional evidence: rs2113389 acts as an expression and methylation quantitative trait locus for *CYP1B1* [21]; among major brain cell types *CYP1B1* expression is highest in excitatory neurons; and *Cyp1b1* expression changes with disease progression in tau-model mice (rTg4510) but not in amyloid-model mice (J20). This is the strongest available argument that the signal at this locus reflects tau biology and not tracer behavior, and it distinguishes this locus from the unreplicated loci described above. This distinction is important because tau-PET is an imaging endophenotype and not clinical AD itself. Consequently, genetic variants may influence regional tau accumulation without conferring measurable risk for AD, suggesting that determinants of tau pathology and overall disease susceptibility are at least partly separable. Nho et al. report that neither rs2113389 nor its linked SNP reached significance in recent large-scale AD case–control GWAS, with inconsistent directions of effect across studies, indicating that this locus is associated with tau-PET burden, not with established genetic susceptibility to clinical AD [21]. Although the functional and imaging evidence strongly supports a role for this locus in tau biology, the absence of association with AD risk means its value as a therapeutic target remains uncertain. These findings therefore provide stronger support for *CYP1B1* as a modifier of tau accumulation than as an established target for disease-modifying therapy, pending further mechanistic and longitudinal studies.

Additionally, *CYP1B1* has been reported to regulate intracellular iron as well as oxidative stress, although that evidence comes from ocular and retinal endothelial tissue and not from brain [59,60], and off-target flortaucipir binding correlates with age-related iron accumulation [61]. A variant that altered regional tissue iron could therefore alter tracer retention without altering tau aggregation, and it would generate an association of exactly the kind reported. Four observations argue against a purely artifactual explanation here. The documented iron-related off-target signal is confined to the basal ganglia and not to the cortical regions in which the *CYP1B1* signal is found. rs2113389 is also associated with cerebrospinal fluid total tau and phosphorylated tau, neither of which is measured with a tracer. The replication stage included AIBL-2, the only contributing cohort not scanned with flortaucipir, which is the most direct available test of a tracer-specific artifact and the one a binding-affinity explanation should not survive; the locus was replicated in a pooled meta-analysis of the five replication cohorts rather than in AIBL-2 alone, so this is suggestive rather than decisive [21]. And *CYP1B1* expression tracks tau pathology in rTg4510 mice but not amyloid pathology in J20 mice [21]. Taken together, these observations favor a tau-biological reading of this locus. However, the approved label for MK-6240 requires that inducers of CYP1A2, including tobacco smoking, be avoided for at least seven days before administration [41,62], which establishes that cytochrome P450 activity can alter the pharmacokinetics of a tau-PET tracer. Both the enzyme and the tracer differ from those at issue here, and an effect on systemic tracer metabolism is not an effect on regional binding, so this does not show that the *CYP1B1* association is artifactual. It does, however, reveal that the class of mechanism the artifact hypothesis invokes is real, which is a further reason to test this locus against a chemically distinct tracer. The same question applies with greater force to every locus in this review for which no comparable orthogonal evidence exists, and for those loci it remains open.

## 7. Protein Phosphorylation and Dephosphorylation Balance

Tau hyperphosphorylation is a defining feature of AD pathology, making protein phosphatase regulation essential for tau homeostasis. One GWAS has identified a genome-wide significant locus in this pathway; the *FYN* signal discussed below derives from a candidate-gene analysis and was not tested genome-wide.

**Figure 2:**
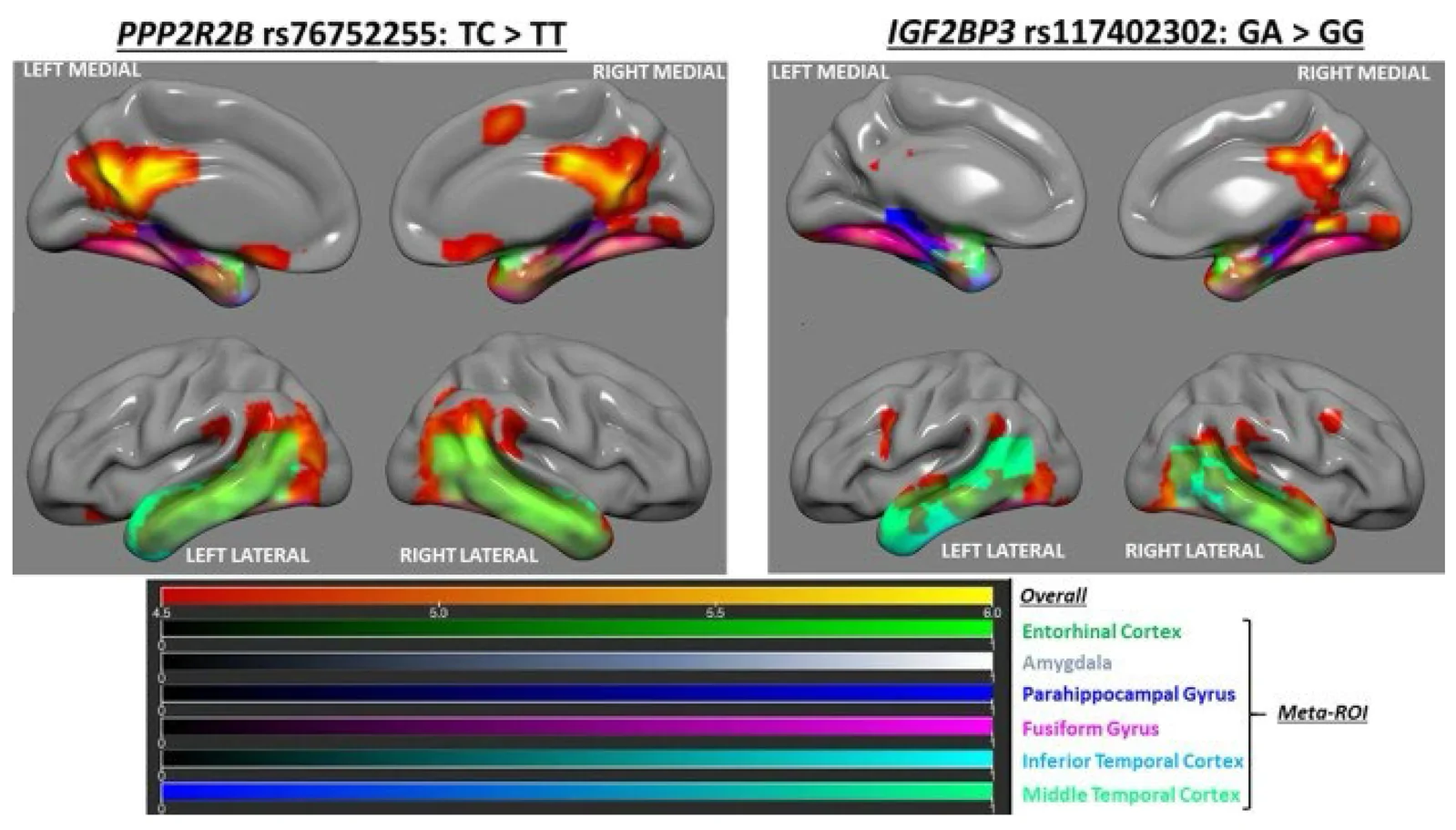
Glass brain surface renderings illustrate results of whole-brain voxel-wise association analyses. Color intensity reflects the strength of the association, with brighter colors on the red-orange-yellow scale indicating progressively stronger signals. Significant clusters associated with the *PPP2R2B* rs76752255-C allele (**left**) and *IGF2BP3* rs117402302 (**right**) are overlaid on the corresponding meta-region of interest (ROI), with individual component regions denoted by distinct color scales. Both variants exhibited increased tau-PET burden, with strongest effects observed in the medial, lateral, and inferior temporal cortices and the posterior cingulate cortex. Adapted from Ramanan, V.K. et al. Variants in *PPP2R2B* and *IGF2BP3* are associated with higher tau deposition. Brain Commun. September 2020, 2(2): fcaa159 with permission under Creative Commons Attribution 4.0 International Public License (https://creativecommons.org/licenses/by/4.0/legalcode) (accessed on 17 July 2026) [22].

*PPP2R2B*, encoding the B55β regulatory subunit of protein phosphatase 2A (PP2A), emerged as a genome-wide significant locus in Ramanan et al. [22]. In the Mayo Clinic Study of Aging, which comprised 754 participants, the authors identified rs76752255 as achieving genome-wide significance (*p* = 9.91 × 10^−9^, β = 0.20) for higher tau deposition. The association remained genome-wide significant when including *APOE* ε4 status, cardiovascular conditions, and global amyloid burden as additional covariates. After accounting for age, sex, genetic principal components, and global amyloid burden, *PPP2R2B* rs76752255 further accounted for 3.4% of the variance in tau-PET. This analysis included no independent replication sample, so the reported effect size should be interpreted cautiously and may be an upper-bound estimate. The minor allele frequency was 1.5% with no minor-allele homozygotes, which precluded any assessment of allele-dosage effects, and a nearby two-SNP haplotype showed a stronger association than the lead variant, leaving the causal variant at this locus uncertain. PP2A is a major serine/threonine phosphatase and the dominant tau phosphatase in human brain, and *PPP2R2B* encodes a B55 regulatory subunit that directs PP2A holoenzyme activity toward particular substrates, including tau [63,64]. Downregulation of PP2A has been shown to promote tau phosphorylation [65,66]. Voxel-wise analyses demonstrated that individuals with the *PPP2R2B* rs76752255-C allele displayed higher tau on a voxel level in an AD-pattern distribution, with predominant clusters in the medial, lateral and inferior temporal and posterior cingulate regions [22] (Figure 2). The spatial distribution of these effects overlaps cortical regions affected early in AD, supporting the biological plausibility of *PPP2R2B* as a modifier of regional tau accumulation, although the association remains unreplicated.

Ramanan et al. reported a second genome-wide significant locus in the same analysis. rs117402302 (*p* = 4.00 × 10^−8^, β = 0.19) is an intergenic variant approximately 11 kb from *IGF2BP3*, which encodes insulin-like growth factor 2 mRNA-binding protein 3 [22]. Unlike *PPP2R2B*, this signal was partially attenuated once global amyloid burden was added as a covariate (*p* = 7.81 × 10^−6^, β = 0.14), and it accounted for a further 1.6% of the variance in tau-PET. Its connection to phosphorylation is indirect: an *IGF2BP3* orthologue binds the 3′ untranslated region of tau mRNA, and insulin-like growth factor signaling has been proposed to influence tau phosphorylation through phosphatidylinositol 3-kinase, Akt, and GSK3β [22]. Because the lead variant is intergenic and assigned to *IGF2BP3* by proximity alone, the gene attribution is provisional, and the locus is grouped in this section on the strength of that indirect link rather than any demonstrated effect on kinase or phosphatase activity.

*FYN* encodes a Src-family tyrosine kinase that phosphorylates tau, making it a plausible modulator of tau aggregation [67]. *FYN* showed a nominal association in Yi et al.’s pathway-based candidate-gene analysis of 146 Korean participants from the KBASE study. Using a targeted approach examining 15 tau pathway-related genes, the authors identified a nominally significant association between *FYN* variants and tau-PET signal in Braak III/IV regions, which include limbic structures implicated in cognitive decline during early AD. The lead *FYN* variant rs57650567 showed a *p*-value of 0.048 for increased tau SUVR. Notably, *FYN* was the weaker of this study’s two lead signals: the strongest association overall was with rs149413552 in *CLU* (clusterin), a well-established Alzheimer’s risk gene involved in lipid transport and immune clearance, which reached *p* = 0.00035 and met the authors’ significance threshold, whereas the *FYN* variant was only nominal. *CLU* therefore warrants at least equal emphasis, and it spans the immune and lipid-transport themes discussed in Section 8 and Section 10. This is a candidate-gene result, not a genome-wide finding, and the SNP-level *p*-value sits at the boundary of nominal significance. It should not be given the same evidential weight as a genome-wide significant, independently replicated locus. Yi et al. also investigated the composite effects of rs57650567 and *APOE* ε4. A stronger effect was found with significantly higher tau deposition in the combined variant group compared to either genetic variant alone (*p* < 2 × 10^−5^) [23]. Additionally, tau was highest in the Braak III/IV regions (*p* < 1 × 10^−5^) in the combined variant group [23].

The phosphorylation pathway is biologically plausible and potentially targetable, with therapeutic implications for modulating kinase and phosphatase activities to restore tau homeostasis. The genetic evidence supporting it from tau-PET GWAS is, however, currently thin: one unreplicated genome-wide significant locus and one nominal candidate-gene association. Neither has been tested against the phenotype-level alternatives described in Section 4, so for both loci the possibility that the variant acts on tracer behavior and not on tau aggregation remains open.

## 8. Immune Response and Neuroinflammation

Neuroinflammation and the subsequent immune response are key factors in AD progression. GWAS of clinically diagnosed AD have identified multiple risk genes involved in immune and neuroinflammatory responses, and a smaller number of these have been tested against tau-PET [68].

Complement Receptor Type 1 (*CR1*) encodes a glycoprotein that mediates interactions between immune cells and their respective targets by binding the complement components C3b and C4b, thereby directing complement-tagged material for clearance [69]. Stage et al. investigated the association of genetic variation in *CR1* with tau deposition using [^18^F]flortaucipir PET imaging in 566 participants from ADNI across three diagnostic subgroups: cognitively normal, mild cognitive impairment (MCI), and dementia [20]. The *CR1* variant, rs3818361, exhibited a significant association, after false discovery rate (FDR) correction (*p*_FDR_ = 0.0464), with tau deposition in the MCI group. Increased tau was seen primarily in the temporal, parietal, and occipital cortices. This suggests that the *CR1*-related immune response influences tau pathology in the earlier stages of AD. This result sits in direct tension with Ramanan et al., who tested 43 variants previously associated with a clinical diagnosis of AD in large case–control studies against tau-PET burden in the Mayo Clinic Study of Aging and reported no nominal associations for any of them [22]. By contrast, Stage et al. identified significant associations between tau-PET burden and several established AD-risk loci, including *CR1*, using an ADNI cohort. The discrepancy may reflect differences in sample (population-based versus ADNI), in the variants tested at each locus, or in analytic approach.

A second immune-related signal comes from *CLU*, which encodes clusterin, a lipid-transport protein that also regulates complement activation. In the KBASE cohort, rs149413552 in *CLU* produced the strongest association reported in that study and met the authors’ significance threshold (*p* = 0.00035) for tau burden in Braak III/IV regions [23]. This is a candidate-gene result in 146 participants and is graded Tier 3 here, but it carries a distinction that no other candidate-gene finding in this review carries: KBASE shares no participants with any other included study, so the finding is not a restatement of an ADNI result. *CR1* and *CLU* are both established Alzheimer risk genes, so their association with tau-PET, if it holds up, would mark a point of contact between the two genetic architectures, and it should be weighed against the argument in Section 11.

## 9. Vesicle Trafficking and Intracellular Transport

Disrupted intracellular trafficking provides a pathway for tau dissemination and accumulation. A candidate-gene analysis has, after false discovery rate correction, implicated a gene affecting vesicle dynamics and protein transport in regional tau deposition.

*BIN1* encodes Bridging Integrator 1, a BAR (Bin/Amphiphysin/Rvs) domain protein that induces membrane curvature and facilitates endocytosis by regulating synaptic vesicle endocytosis and neurotransmitter release through its interactions with dynamin and synaptojanin [70,71]. Franzmeier et al. reported that carriers of the *BIN1* rs744373 risk allele showed increased [^18^F]flortaucipir uptake across Braak stage II–VI regions in 89 older adults without dementia, while no association was observed with amyloid PET, suggesting a preferential relationship between *BIN1* variation and tau pathology [24]. Stepwise regression analyses by Stage et al. found *BIN1* rs6733839 to be significantly associated with greater tau-PET signals (*p*_FDR_ = 0.0464), supporting a possible relationship between *BIN1* genetic variation and tau deposition [20]. *BIN1* deficiency in neuronal cultures impairs tau secretion and promotes intracellular tau accumulation, while *BIN1* variants associated with AD risk reduce protein expression and disrupt endosomal trafficking [72]. This disruption may facilitate tau propagation between neurons through impaired clearance and altered vesicle dynamics at synaptic terminals, although the PET associations themselves do not establish a direct mechanism. Stage et al. report that voxel-wise analysis of *BIN1* rs6733839 showed no family-wise-error-corrected association with tau deposition, in contrast to the *CR1* and *CASS4* variants in the same model. The regional interpretation offered here therefore rests on the meta-ROI regression alone.

## 10. Lipid Transport and Membrane Biology

*APOE* is the major apolipoprotein in brain, responsible for cholesterol transport and membrane maintenance [73]. Beyond these lipid-related effects, *APOE* ε4, the most frequently reported genetic determinant of tau-PET burden, has been reported to interact with tau protein and influence its clearance through isoform-specific differences in binding or degradation mechanisms [17].

The association between *APOE* ε4 and tau accumulation is not uniformly reproducible across cohorts. Nho et al. reported a genome-wide significant association between ε4 and elevated tau in medial temporal, parietal, and posterior cingulate regions [19,21,22]; allele-dose relationships, however, were not formally reported in the studies reviewed here. The strength and independence of the *APOE*–tau association vary substantially depending on cohort characteristics and amyloid burden. In the population-based Mayo Clinic Study of Aging, in which roughly 87% of participants were cognitively unimpaired, *APOE* ε4 showed only a marginal association with tau-PET burden (*p* = 0.06), and in Guo et al. *APOE* reached genome-wide significance only when *APOE* ε4 was omitted as a covariate, suggesting that *APOE* may influence the detection of additional tau-associated loci [19,22]. The *APOE* effect on tau-PET therefore appears robust in amyloid-positive and clinically enriched samples and attenuated in unimpaired population-based samples, a pattern consistent with an effect that is substantially amyloid-conditional. Both the entorhinal and inferior temporal associations between *APOE* ε4 and tau in the Mayo cohort were fully attenuated after adjustment for global amyloid burden [22]. Statistical attenuation and biological dependence should, however, be kept separate here. Loss of significance after adjustment for amyloid burden is consistent with amyloid lying on the causal path from *APOE* ε4 to tau, and it is equally consistent with amyloid burden acting as a proxy for disease stage, with collider bias when amyloid positivity is conditioned on in a sample selected partly on cognition, and with a simple loss of power once a strongly correlated covariate enters the model. None of the studies reviewed here carried out a formal mediation analysis with its identifying assumptions stated and probed, so the amyloid-conditional reading is the most economical account of the pattern and not a demonstration of mediation. In contrast, evidence for amyloid-independent genetic effects on tau is supported by findings at non-*APOE* loci: *PPP2R2B* rs76752255 remained genome-wide significant after adjustment for global amyloid burden, and rs2113389 at *CYP1B1-RMDN2* showed additive but not interactive effects with amyloid positivity.

Additionally, studies suggest *APOE* ε4 may promote tau hyperphosphorylation by activating GSK3β and p38 MAPK signaling pathways, though the direct versus indirect nature of these molecular interactions requires further investigation [74]. These potential tau-related mechanisms, combined with *APOE* ε4’s established effects on membrane integrity and neuroinflammation, may create convergent pathways that collectively promote tau pathology.

Two further loci reported by Stage et al. are listed under this heading in Table 4. rs3764650 in *ABCA7*, which encodes an ATP-binding cassette transporter involved in lipid efflux and phagocytosis, was associated with tau-PET signal in the cognitively normal subgroup, and rs7274581 in *CASS4* was associated in the mild cognitive impairment and dementia subgroups [20]. Both are candidate-gene results obtained within diagnostic subgroups of a single ADNI sample, and neither has been replicated. *CASS4* encodes a Cas-family scaffolding protein with roles in focal adhesion and cytoskeletal organization rather than in lipid transport; it is grouped here because it was reported alongside the lipid-related loci and not because it shares their mechanism.

The loci discussed in Section 5, Section 6, Section 7, Section 8, Section 9 and Section 10 are summarized in Table 4, grouped by the biological process to which each has been attributed and graded by the evidence tier assigned in Table 2. The distribution is uneven: one locus carries a pre-specified independent replication, and the remainder rest on a single analysis, most of them in overlapping samples.

## 11. Preliminary Evidence That the Genetic Architecture of Tau-PET Is Partly Separable from That of Clinical Alzheimer’s Disease

The studies reviewed here provide preliminary evidence that the genetic architecture of tau-PET signal is at least partly distinct from that of clinically diagnosed AD. The evidence is indirect, and the strength of the claim should be read against the qualifications set out at the end of this section.

Two studies support this reading through different analyses. Ramanan et al. tested 43 variants previously associated at genome-wide significance with a clinical diagnosis of probable AD and found that none was even nominally associated with tau-PET burden [22]. Nho et al. found that rs2113389, the strongest and, to date, only replicated locus in this literature, was not significantly associated with AD in recent large-scale case–control GWAS, with inconsistent directions of effect across those studies [21].

Stage et al. is the exception. This study reported associations between tau-PET signal and several established Alzheimer’s risk loci, including *CR1*, *BIN1*, *ABCA7*, and *CASS4* [20]. Whether this reflects the ADNI sample, the variants selected at each locus, or the stepwise analytic approach is unresolved, and the discrepancy with Ramanan et al. has not been addressed in this literature.

Three qualifications limit how far this argument can be taken. First, no formal genetic correlation has been estimated between tau-PET GWAS summary statistics and case–control AD GWAS, so separability has been inferred from the failure of individual variants to cross significance thresholds and has not been measured. Second, the tau-PET samples are small, so the failure of an established AD risk variant to reach even nominal significance against tau-PET is weak evidence that no effect exists; at these sample sizes an absence of evidence is close to uninformative for variants of modest effect. Third, the studies contributing to this argument overlap in the cohorts they analyze, so their negative results are not independent of one another. Stage et al. and the *CLU* result from KBASE both point in the opposite direction. What the current data support is partial separability, offered here as a hypothesis to be tested by the analyses described in Section 14 and not as an established property of the two architectures.

Two consequences follow if this interpretation is correct. First, it strengthens the case for studying tau-PET as an endophenotype and not as a proxy for disease risk, because the loci it identifies are not the loci that case–control studies find. Second, it complicates the therapeutic argument advanced by these studies and by this review. A variant that governs how much tau a person accumulates but does not predict whether that person develops dementia is not a straightforward drug target. In the primary studies and in reviews of them, these loci are described as therapeutic targets with an enthusiasm that currently exceeds the evidence.

## 12. Reference Standards for Tau-PET and Their Implications for Imaging Genetics

Section 4 described what tau-PET measures. A separate question is what any tau biomarker should be measured against, because the reference standard chosen determines what a reported association can be taken to mean. Diagnostic accuracy studies are traditionally evaluated through sensitivity, specificity, positive predictive value, and negative predictive value relative to an independent reference standard. The STARD (Standards for Reporting Diagnostic Accuracy Studies) 2015 guidelines emphasize the importance of clearly defining both the index test and the reference standard when evaluating diagnostic performance [75]. Investigators should describe participant selection, report diagnostic thresholds, and account for indeterminate or missing results. While STARD does not dictate how diagnostic studies must be conducted, it provides a framework for evaluating whether sufficient methodological rigor exists to support claims regarding diagnostic performance.

Application of these principles to AD biomarkers presents unique challenges because there is no universally applicable in vivo reference standard. Neuropathological examination at autopsy remains the definitive method for confirming the density, distribution, and molecular composition of neurofibrillary tangles and amyloid plaques. However, autopsy confirmation is rarely available in large prospective imaging studies because participants may survive many years following biomarker assessment, resulting in relatively few imaging-pathology correlation studies. Consequently, many investigations substitute clinical diagnosis or longitudinal clinical progression as surrogate reference standards.

Both surrogates introduce errors of their own. Clinical diagnosis is the more common of the two and is imprecise. In a large autopsy-confirmed cohort from the National Alzheimer’s Coordinating Center (NACC), sensitivity of the clinical diagnosis of probable AD ranged from 70.9% to 87.3%, while specificity ranged from only 44.3% to 70.8%, depending on the neuropathologic threshold applied. Among 271 subjects clinically diagnosed as not having probable or possible AD, 107 cases (39%) nevertheless met histopathologic criteria for AD at autopsy, demonstrating substantial false-negative clinical classification [76]. Conversely, among subjects clinically diagnosed as probable AD, 88 cases failed to meet neuropathologic thresholds for AD and instead demonstrated alternative or mixed pathologies including frontotemporal lobar degeneration, Lewy body disease, cerebrovascular disease, hippocampal sclerosis, and progressive supranuclear palsy [76]. These figures show the extent of clinicopathologic overlap among neurodegenerative dementias and the limits of clinical diagnosis as a validation standard. The second surrogate, longitudinal clinical progression, is no better, because it does not account for cognitive reserve, which is the brain’s capacity to maintain functional performance despite underlying structural damage [77]. Two patients with identical pathological burdens may exhibit different trajectories of cognitive decline, rendering clinical tracking an inconsistent benchmark for biological disease burden. The 2026 approval of MK-6240 shows how far this reaches into clinical practice. Diagnostic performance was established against a reference standard built from cognitive status and amyloid-PET findings and not against neuropathology, with positive percent agreement of 80% to 88% across readers in one blind-read study and 68% to 82% in the second, alongside negative percent agreement of 93% to 99% [41]. Those figures describe agreement with a composite clinical and biomarker construct, so they cannot be read as sensitivity against tau pathology itself. The label states in addition that safety and effectiveness have not been established for non-Alzheimer tauopathies, which is the regulatory form of the limitation described in Section 4.

Future studies incorporating prospective autopsy validation, standardized diagnostic accuracy methodologies, and transparent adherence to STARD reporting criteria will be essential for determining the clinical utility and diagnostic performance of tau biomarkers across the Alzheimer’s disease continuum. For imaging genetics, the immediate consequence is narrower. A locus associated with tau-PET cannot be validated against a clinical diagnosis without inheriting the error of that diagnosis, with sensitivity and specificity in the range reported above. This is one reason the apparent separation between tau-PET genetics and the genetics of clinically diagnosed AD, described in Section 11, is difficult to interpret: part of the discordance may lie in the imprecision of the case definition used in case–control studies and not in the biology of the two phenotypes.

## 13. Limitations of Tau-PET GWAS

Although tau-PET GWAS have identified numerous loci associated with regional tau-PET signal, several limitations should be considered when interpreting these findings. Primarily, most studies remain modest in size compared with contemporary GWAS in other complex diseases. Participants in these cohorts are drawn largely from ADNI and other relevant patient pools, resulting in a predominantly European ancestry population [21]. These cohorts are not merely similar but substantially overlapping: ADNI contributes participants to Guo et al., Stage et al., Franzmeier et al., and the discovery meta-analysis of Nho et al., while the Mayo Clinic Study of Aging is analyzed by Ramanan et al. and also contributes to the replication stage of Nho et al. Only the Korean KBASE cohort is fully independent of the others. Convergence of findings across these reports therefore cannot be treated as replication in independent samples. Multiethnic cohorts and sex-stratified analyses have largely not been undertaken [19,20,21,22,23]. Consequently, the generalizability of identified loci to diverse populations remains uncertain. Sample size also constrains reliability, not only generalizability. At these sample sizes only comparatively large effects can cross the genome-wide significance threshold, so variants that do cross it are subject to winner’s curse and their reported effect sizes should be treated as upper bounds, while true modest effects are likely to have been missed. Replication status, not the *p*-value alone, should therefore govern how much weight each locus is given. A further constraint is the boundary of this review. A genome-wide analysis of tau-PET signal has been posted as a preprint and reports additional loci, but it has not completed peer review and was excluded under the criteria set out in Section 2 [78]. The peer-reviewed literature summarized here is therefore not the whole of the reported literature, and the tiers in Table 2 grade what has passed review and not everything that has been claimed.

Another point of consideration is the use case of GWAS to identify genetic associations for measured phenotypes and not to analyze mechanisms. In tau-PET GWAS, the phenotype under investigation is tracer uptake, not directly measured neuropathological tau burden. Therefore, genetic associations may reflect influences on tau aggregation, tracer binding characteristics, regional neurodegeneration, tissue composition, or other biological processes contributing to PET signal [21]. A related concern is that genetic associations with tau-PET uptake could reflect variation in tracer behavior, not underlying tau pathology. This issue is particularly relevant for loci such as *CYP1B1*, a cytochrome P450 family member whose biological functions include oxidative metabolism and regulation of lipid and steroid pathways, with no known direct role in tau aggregation itself. Nho et al. addressed this possibility by demonstrating that the lead *CYP1B1*-*RMDN2* locus variant (rs2113389) was associated not only with flortaucipir PET signal but also with cerebrospinal fluid total tau and phosphorylated tau levels, suggesting that the locus influences tau-related biology beyond PET signal alone [21]. Furthermore, *CYP1B1* expression tracked with tau pathology progression in tauopathy mouse models but not amyloid models, providing evidence that the locus reflects biological processes related to tau pathology, not solely nonspecific tracer effects [21]. This limitation is further compounded by the incomplete understanding of the biological significance of tau accumulation and tau-PET signal in relation to neurodegeneration and cognitive impairment. Although elevated tau-PET uptake is strongly associated with disease progression and clinical severity, the extent to which PET-detected tau directly mediates neuronal injury versus serving as a correlated biomarker of broader pathophysiologic processes remains unresolved. Therefore, the identification of genetic determinants of tau-PET signal should not automatically be interpreted as establishing genetic determinants of neurodegeneration, neuronal loss, or cognitive decline. The clearest worked example of this problem is the *CYP1B1-RMDN2* locus, where an iron-mediated effect on tracer retention is a coherent alternative to an effect on tau aggregation. That argument is set out in Section 6, and the general form of it is set out in Section 4. It applies with greater force to every locus in this review for which no comparable orthogonal evidence exists, which is most of them.

## 14. Future Directions

Several concrete steps follow from the limitations set out above and would do more to advance this field than the discovery of further unreplicated loci.

Cross-tracer replication should become a standard requirement. Because the phenotype in these studies is tracer uptake and not tau itself, any locus that acts on tracer chemistry and not on tau biology will replicate readily across flortaucipir cohorts and fail across chemically distinct tracers. Testing whether a locus survives a switch to a second-generation tracer such as MK-6240, RO948, or PI-2620 is therefore not merely a robustness check but a direct discriminator between the two interpretations. Nho et al. provide the only example to date [21]. Cross-tracer replication is more practicable than it was when the studies reviewed here were designed. A second tau tracer received regulatory approval in the United States in August 2026 [62], and the CLARiTI consortium is collecting standardized MK-6240 tau-PET, MRI, and plasma biomarkers on 2000 participants across the Alzheimer’s Disease Research Center network [79]. Because that network also supplies genome-wide genotyping and long-term autopsy follow-up, CLARiTI is positioned to deliver at once the three things this literature most needs: a second tracer, cohorts independent of ADNI, and eventual neuropathological validation of the phenotype itself.

The phenotype itself can also be broadened. Tau-PET is one of several quantitative PET measures obtainable in these cohorts, and glucose metabolism and molecular calcification carry information about the aging brain that tau tracers do not; both have been proposed as contributors to risk stratification in mild cognitive impairment [80]. Each defines a continuous phenotype that could be carried to genome-wide analysis in the same participants, and a study acquiring more than one tracer would allow a locus to be tested for specificity to tau instead of being reported against a single measure. That design would also let the confounders described in Section 4 be modeled instead of assumed away, because a candidate locus could be tested for association with tau-PET signal after adjustment for regional mineralization measured with [^18^F]NaF [80].

Sample size and cohort diversity must both increase, and they are not the same problem. Current tau-PET GWAS are small enough that only large effects can cross the genome-wide threshold, so the loci that do cross it are systematically inflated; and they are drawn overwhelmingly from ADNI, so apparent convergence across studies is not independent replication [20,21,22,23,81]. Consortium-scale aggregation addresses the first; fully independent cohorts, including the Korean KBASE cohort and the Alzheimer’s Biomarker Consortium–Down Syndrome, address the second [23,82]. Neither substitutes for the other.

Longitudinal tau-PET phenotypes remain almost entirely unexploited. Every study reviewed here is cross-sectional and therefore tests genetic influences on tau burden at a single moment and not on the rate at which tau accumulates. Several of the contributing cohorts have serial tau-PET; the rate of change is the phenotype most closely tied to the biology of propagation, and most relevant to trial design.

Genetic resistance to tau deposition warrants the same attention that susceptibility has received. Ramanan et al. framed the original question as one of susceptibility versus resistance to tau deposition, but the literature that followed has reported almost exclusively risk-increasing variants. Loci associated with lower tau burden despite advanced age or amyloid positivity are of equal mechanistic and greater therapeutic interest and are unlikely to be found unless they are looked for [22].

Finally, the separability of tau-PET genetics from AD genetics should be tested directly and not inferred from scattered negative results. Formal genetic correlation analysis between tau-PET GWAS summary statistics and case–control AD GWAS, once tau-PET sample sizes permit it, would test this separability directly. The comparison should not stop at two phenotypes. The genome-wide literature on amyloid-PET has been reviewed separately [16], and a three-way comparison of the genetic architectures of tau-PET, amyloid-PET, and clinically diagnosed AD is the question this field is ultimately asking.

## 15. Conclusions

Tau-PET imaging combined with GWAS has identified genetic variants associated with regional tau-PET signal and has provided insight into biological pathways potentially related to tau accumulation and neurodegeneration. The genetic architecture of tau-PET signal appears at least partly separable from that of clinically diagnosed AD: variants that predict a clinical diagnosis largely do not predict tau-PET burden, and the one replicated tau-PET locus is not an Alzheimer’s risk variant. This separability has not been measured directly, and the samples supporting it are small and overlapping, so it should be read as a hypothesis and not as a settled result. It is nonetheless a reason to take tau-PET seriously as an endophenotype. It is also a reason for restraint about the therapeutic claims made for these loci, in the primary studies and in reviews such as this one.

Despite substantial advances in tau imaging and genetics, important questions remain regarding the biological significance of tau accumulation, the causal role of tau in neurodegeneration, and the extent to which tau-PET accurately reflects underlying neuropathology across disease stages. Although tau-PET has emerged as a valuable research tool and has demonstrated meaningful associations with clinical and genetic factors, ongoing challenges involving tracer specificity, neuropathological validation, diagnostic accuracy, and reference standard selection warrant continued investigation. Future studies integrating genetics, longitudinal imaging, fluid biomarkers, and postmortem neuropathology will be essential to determine whether tau-related biomarkers primarily provide mechanistic insight, clinical utility, or both.

## Figures and Tables

**Table 1:** Genome-wide and candidate-gene association studies of tau-PET imaging in Alzheimer’s disease: study characteristics and reported loci.

Study Name	Population	Functional Imaging	Type of Analysis	Implicated Genes for Tau-PET Signal and Statistical Significance
Guo et al. [19]	European, non-demented individuals (*N* = 543); ADNI	AV1451 PET	GWAS of brain tau load as measured by [^18^F]AV-1451 PET	rs56298435 in *ZBTB20* (*p* = 8.35 × 10^−10^, β = 0.31)
				rs150532 in *EYA4* (*p* = 1.90 × 10^−8^, β = 0.26)
				*APOE* association reached genome-wide significance when *APOE* ε4 was not adjusted.
Nho et al. [21]	Twelve cohorts (*N* = 3046): discovery 7 cohorts (*N* = 1446); replication 5 cohorts (*N* = 1600)	tau-PET	GWAS of cortical tau quantified by PET	rs2113389 SNP in *CYP1B1*-*RMDN2* locus → 4.3% of cortical tau variation; replicated in 5 independent cohorts (Z = 3.83, *p* = 1.26 × 10^−4^, concordant direction)
				rs429358 in *APOE* ε4 → 3.6% of cortical tau variation
Ramanan et al. [22]	Age over 50 (mean age 72.4 years, 54.6% men, 87.6% cognitively unimpaired) from the population-based Mayo Clinic Study of Aging (*N* = 754)	AV-1451 (^18^F-flortaucipir) tau-PET	GWAS of AV-1451 (^18^F-flortaucipir) tau-PET burden in Alzheimer’s signature brain regions, covarying for age, sex, and ancestry	rs76752255 in *PPP2R2B* (protein phosphatase 2 regulatory subunit B; tau phosphorylation regulator) (*p* = 9.91 × 10^−9^, β = 0.20)
				rs117402302 near *IGF2BP3* (insulin-like growth factor 2 mRNA-binding protein 3) (*p* = 4.00 × 10^−8^, β = 0.19)
				*MAPT* locus (tau gene): 3 SNPs showed nominal associations with tau-PET burden
				*APOE* ε4 allele: marginal/nonsignificant association (*p* = 0.06, β = 0.07)
Stage et al. [20]	ADNI-2 and ADNI-3: 352 cognitively normal, 160 mild cognitive impairment, 54 dementia (*N* = 566)	[^18^F]flortaucipir SUVR (meta–region of interest)	Step-wise regression with log-transformed [^18^F]flortaucipir meta-region of interest SUVR as the outcome measure and genetic variants, age, sex, and apolipoprotein E (*APOE*) ε4 as predictors	rs429358 in *APOE* ε4: Significant association with tau deposition across all disease stages (cognitively normal, mild cognitive impairment, and dementia) (*p* < 0.05)
				rs3764650 in *ABCA7* (*p* < 0.05 in cognitively normal)
				rs3818361 in *CR1* (*p* < 0.05 in mild cognitive impairment)
				rs6733839 and rs744373 in *BIN1* (*p* < 0.05 in mild cognitive impairment and dementia)
				rs7274581 in *CASS4* (*p* < 0.05 in mild cognitive impairment and dementia)
Yi et al. [23]	Korean AD continuum cohort (Korean Brain Aging Study for the Early Diagnosis and Prediction of Alzheimer’s Disease; KBASE) (*N* = 146)	^18^F-AV-1451 PET	Pathway-based candidate gene association analysis using PLINK with additional voxel-wise analysis conducted using SPM12. (PLINK is a tool set for whole-genome association and population-based linkage analyses on tau deposition; SPM12: Statistical Parametric Mapping 12)	rs149413552 in *CLU*: showed the strongest association indicating a significant correlation with tau burden in the Braak III/IV ROI (*p* = 0.00035)
				rs57650567 in *FYN*: showed a nominal association with tau burden in the Braak III/IV ROI (*p* = 0.048)
Franzmeier et al. [24]	Non-demented, older adults from ADNI (40 MCI, 49 cognitively normal) (*N* = 89)	[^18^F]AV-1451 (flortaucipir) tau-PET; [^18^F]AV-45 amyloid-PET also assessed	Candidate SNP association analysis using ANCOVA and regression models to evaluate the association between *BIN1* variants and regional/global tau-PET SUVR. Regional analyses examined Braak stage–based ROIs, and mediation analysis tested whether tau burden mediated memory impairment.	Carriers of the *BIN1* rs744373 risk allele showed significantly higher global tau-PET burden (*p* = 0.007) after adjustment Elevated tau across Braak II–VI regions; tau mediated the association between the *BIN1* risk allele and memory impairment (*p* = 0.016)

**Table 2:** Cohort composition, sample overlap, replication status, and evidence tier of the tau-PET GWAS and candidate-gene studies included in this review. Tier 1, genome-wide significant and replicated in an independent sample; Tier 2, genome-wide significant but unreplicated; Tier 3, significant in a pre-specified candidate-gene analysis, with correction for multiple testing where more than one variant was tested; Tier 4, nominal association only (*p* < 0.05, uncorrected). Tiers are assigned to individual results and not to studies as a whole, and the same definitions are applied to genome-wide, gene-based, candidate-gene, and nominal findings; a study reporting results at more than one tier therefore appears at more than one tier. The scheme was created for this review and does not correspond to GRADE, the Oxford Centre for Evidence-Based Medicine levels, or any other established grading system, for the reasons given in Section 2. ADNI participants contribute to four of the six reports and Mayo Clinic Study of Aging participants to two, so apparent convergence of findings across these studies does not amount to replication in independent samples.

Study	Cohort(s)	Overlap with Other Included Studies	Replication	Evidence Tier
Guo et al. [19]	ADNI (*N* = 543)	ADNI shared with Stage, Franzmeier, and the Nho discovery set	None reported	Tier 2: genome-wide significant, unreplicated
Nho et al. [21]	Discovery: ADNI, ADNI-DoD, IMAS, A05, A4, HABS, UPitt ADRC (*N* = 1446). Replication: MCSA, MAP–Knight ADRC, AIBL-1, AIBL-2, BACS (*N* = 1600)	Discovery shares ADNI with Guo, Stage, and Franzmeier; replication shares MCSA with Ramanan	Yes: 5 independent cohorts, concordant direction (Z = 3.83, *p* = 1.26 × 10^−4^). Supported by eQTL, meQTL, single-nucleus RNA-seq, and tau- vs. amyloid-model mouse data	Tier 1: replicated genome-wide significant with orthogonal functional support
Ramanan et al. [22]	Mayo Clinic Study of Aging (*N* = 754)	MCSA also contributes to the Nho replication set	None: no replication sample	Tier 2: genome-wide significant, unreplicated
Stage et al. [20]	ADNI-2/ADNI-3 (*N* = 566)	ADNI shared with Guo, Franzmeier, and the Nho discovery set	None reported	Tier 3: candidate-gene; FDR-corrected, not tested genome-wide
Yi et al. [23]	KBASE, Korea (*N* = 146)	None: the only fully independent cohort in this review	None reported	Tier 3 for *CLU* (rs149413552, *p* = 0.00035, the study’s significant lead); Tier 4 for *FYN* (nominal, *p* = 0.048)
Franzmeier et al. [24]	ADNI (*N* = 89)	ADNI shared with Guo, Stage, and the Nho discovery set	None reported	Tier 3: significant candidate-gene association (*BIN1* rs744373) with tau-PET; not a genome-wide discovery study

**Table 3:** Methodological features of the included studies. Differences in tracer, phenotype definition, cohort setting, ancestry, covariate adjustment, significance threshold, and replication strategy are among the most likely sources of discordant findings across this literature and should be read alongside the evidence tiers in Table 2. Abbreviations: ADNI, Alzheimer’s Disease Neuroimaging Initiative; ANCOVA, analysis of covariance; CSF, cerebrospinal fluid; eQTL, expression quantitative trait locus; FDR, false discovery rate; KBASE, Korean Brain Aging Study for the Early Diagnosis and Prediction of Alzheimer’s Disease; MCI, mild cognitive impairment; meQTL, methylation quantitative trait locus; ROI, region of interest; SUVR, standardized uptake value ratio.

Study	Tracer and Tau-PET Phenotype	Cohort Setting and Ancestry	Covariates in the Primary Model	Analytic Approach and Threshold	Replication Strategy
Guo et al. [19]	[^18^F]AV-1451 (flortaucipir); regional and global tau SUVR	ADNI; clinic-based; European ancestry; non-demented, from normal cognition to MCI	Age, sex, ancestry principal components, *APOE* ε4; a parallel model omitted *APOE* ε4	Single-cohort GWAS; *p* < 5 × 10^−8^; mediation analysis of tau-PET on cognition	None reported; concordant direction in CSF and plasma p-tau in the same participants
Nho et al. [21]	Flortaucipir in the discovery cohorts, with a chemically distinct tracer in part of the replication stage; cortical meta-ROI SUVR and voxel-wise maps	Twelve cohorts (discovery *N* = 1446; replication *N* = 1600); mixed clinic-based and research cohorts; European ancestry	Age, sex, two ancestry principal components, diagnosis, and *APOE* ε4 status; the voxel-wise ANCOVA additionally controlled for amyloid positivity	Fixed-effects discovery meta-analysis; *p* < 5 × 10^−8^; heterogeneity assessed (I^2^ = 27.8%)	Pre-specified replication in five independent cohorts, concordant direction (Z = 3.83, *p* = 1.26 × 10^−4^); eQTL, meQTL, single-nucleus RNA-seq, and tau- versus amyloid-model mouse data
Ramanan et al. [22]	AV-1451 (flortaucipir); Alzheimer-signature meta-ROI SUVR and voxel-wise maps	Mayo Clinic Study of Aging; population-based; predominantly European ancestry; 87.6% cognitively unimpaired	Age, sex, ancestry principal components; sensitivity models added *APOE* ε4, cardiovascular conditions, and global amyloid burden	GWAS at *p* < 5 × 10^−8^; separate candidate analyses of 31 *MAPT* variants (*p* < 6.58 × 10^−4^) and of 43 established AD risk variants	No replication sample analyzed
Stage et al. [20]	[^18^F]flortaucipir; log-transformed meta-ROI SUVR and voxel-wise maps	ADNI-2 and ADNI-3; clinic-based; 352 cognitively normal, 160 MCI, 54 dementia	Age, sex, *APOE* ε4	Stepwise regression of variants at the top 20 AD risk genes; FDR correction; voxel-wise analyses family-wise-error corrected; not tested genome-wide	None reported; results presented separately within diagnostic subgroups
Yi et al. [23]	^18^F-AV-1451; Braak-stage ROI SUVR with voxel-wise analysis in SPM12	KBASE, Korea; Korean ancestry; AD continuum; the only cohort with no participant overlap with the others	Age, sex, *APOE* ε4 carrier status	Pathway-based candidate-gene analysis of 15 tau-pathway genes in PLINK; study-defined threshold; not tested genome-wide	None reported
Franzmeier et al. [24]	[^18^F]AV-1451; global and Braak-stage ROI SUVR; [^18^F]AV-45 amyloid-PET also acquired	ADNI; clinic-based; 40 MCI and 49 cognitively normal (*N* = 89); predominantly European ancestry	Age, sex, diagnosis, *APOE* ε4	Single-variant candidate analysis of *BIN1* rs744373 by ANCOVA and regression, with mediation analysis for memory	None reported; no association with amyloid-PET in the same participants

**Table 4:** Reported genetic determinants of tau-PET signal, grouped by the biological process to which each locus has been attributed and graded by the evidence tier defined in Section 2 and applied in Table 2. Full statistics for each association are given in Table 1. The groupings are organizational and do not imply that the genes within a group act through a shared molecular pathway. Only *CYP1B1*-*RMDN2* reaches Tier 1 on the strength of a pre-specified independent replication; every other locus rests on a single analysis, and most of the contributing cohorts overlap, so the table should be read as a map of what has been reported and not as a set of independently established findings. * *APOE* ε4 is placed at Tier 1 based on the genome-wide significant, independently replicated association reported by Nho et al., with the Guo and Stage results treated as supporting rather than qualifying evidence; its association was marginal in the population-based Mayo Clinic Study of Aging, and the amyloid-conditional character of the effect is discussed in Section 10.

Biological Process	Locus	Lead Variant	Study	Evidence Tier
Transcriptional and chromatin regulation (Section 5)	*ZBTB20*	rs56298435	Guo et al. [19]	Tier 2
	*EYA4*	rs150532	Guo et al. [19]	Tier 2
Microtubule dynamics and oxidative stress (Section 6)	*CYP1B1-RMDN2*	rs2113389	Nho et al. [21]	Tier 1
	*MAPT*	rs1467967	Ramanan et al. [22]	Tier 4
Phosphorylation and dephosphorylation balance (Section 7)	*PPP2R2B*	rs76752255	Ramanan et al. [22]	Tier 2
	*IGF2BP3*	rs117402302	Ramanan et al. [22]	Tier 2
	*FYN*	rs57650567	Yi et al. [23]	Tier 4
Immune response and neuroinflammation (Section 8)	*CR1*	rs3818361	Stage et al. [20]	Tier 3
	*CLU*	rs149413552	Yi et al. [23]	Tier 3
Vesicle trafficking and intracellular transport (Section 9)	*BIN1*	rs744373; rs6733839	Franzmeier et al. [24]; Stage et al. [20]	Tier 3
Lipid transport and membrane biology (Section 10)	*APOE* ε4 *	rs429358	Nho et al. [21]; Guo et al. [19]; Stage et al. [20]	Tier 1
	*ABCA7*	rs3764650	Stage et al. [20]	Tier 3
	*CASS4*	rs7274581	Stage et al. [20]	Tier 3

## Data Availability

No new data were created or analyzed in this study. Data sharing is not applicable to this article.
